# Hospital-at-home and beyond: experiences of patients, caregivers and general practitioners with out-of-hospital care for moderate-to-severe lower respiratory tract infections in older adults—a qualitative study

**DOI:** 10.1093/ageing/afag144

**Published:** 2026-07-10

**Authors:** Rianne M C Pepping, Isis Vink, Rick Roos, Maarten O van Aken, Frederiek van den Bos, Gerrit-Dirk de Loor, Hanneke Borgdorff, Rimke C Vos, Cees van Nieuwkoop

**Affiliations:** Department of Public Health and Primary Care; Health Campus The Hague, Leiden University Medical Center, Hippocratespad 21 Building 3, Sixth Floor, Leiden 2300 RC, The Netherlands; Internal Medicine, Haga Hospital, Den Haag, Zuid-Holland, The Netherlands; Department of Public Health and Primary Care; Health Campus The Hague, Leiden University Medical Center, Hippocratespad 21 Building 3, Sixth Floor, Leiden 2300 RC, The Netherlands; Department of Public Health and Primary Care; Health Campus The Hague, Leiden University Medical Center, Hippocratespad 21 Building 3, Sixth Floor, Leiden 2300 RC, The Netherlands; Internal Medicine, Haga Hospital, Den Haag, Zuid-Holland, The Netherlands; Department of Public Health and Primary Care; Health Campus The Hague, Leiden University Medical Center, Hippocratespad 21 Building 3, Sixth Floor, Leiden 2300 RC, The Netherlands; Internal Medicine, Haga Hospital, Den Haag, Zuid-Holland, The Netherlands; Department of Internal Medicine, Section Gerontology and Geriatrics, Leiden Universitair Medisch Centrum, Leiden, Zuid Holland, The Netherlands; Florence, Den Haag, Zuid-Holland, The Netherlands; Department of Internal Medicine, Section Gerontology and Geriatrics, Leiden Universitair Medisch Centrum, Leiden, Zuid Holland, The Netherlands; Department of Public Health and Primary Care; Health Campus The Hague, Leiden University Medical Center, Hippocratespad 21 Building 3, Sixth Floor, Leiden 2300 RC, The Netherlands; Department of Public Health and Primary Care; Health Campus The Hague, Leiden University Medical Center, Hippocratespad 21 Building 3, Sixth Floor, Leiden 2300 RC, The Netherlands; Internal Medicine, Haga Hospital, Den Haag, Zuid-Holland, The Netherlands

**Keywords:** older adults, hospital at home, lower respiratory tract infection, qualitative research, patient and healthcare provider experiences

## Abstract

**Introduction:**

Hospital-at-home (HaH) may help address rising healthcare demands in ageing populations by providing hospital-level care outside the hospital. In the Netherlands, an integrated care pathway (ICP) ‘The Hague Respiratory Tract Infection Care Bridge’, was developed for adults aged ≥65 years with moderate-to-severe lower respiratory tract infections, offering care through HaH, hospital-at-nursing-home (HaNH) or hospital admission. This study explored experiences with this ICP among the different care settings from the perspectives of patients, informal caregivers and general practitioners (GPs).

**Methods:**

Semi-structured interviews were conducted with participants across the 3 pathways, including 34 patients, 33 informal caregivers and 7 HaH GPs. Data were analysed using deductive thematic analysis guided by the Consolidated Framework for Implementation Research.

**Results:**

Three themes emerged from patient and informal caregiver interviews: matching care to needs, shared decision-making and communication, and organisational collaboration. HaH and HaNH were generally seen as positive alternatives to hospitalisation, although feasibility depended on patients’ and caregivers’ physical and emotional circumstances. Overall satisfaction with care was high. Two themes were identified from GP interviews: practical feasibility and engagement in the HaH-pathway. Overall, GPs supported out-of-hospital treatments, but faced barriers regarding unclear responsibilities, minimal involvement in triage and fragmented communication across providers.

**Conclusion:**

Delivering hospital-level care outside the hospital for older adults requires context-sensitive, individualised approaches that align with patients’ and informal caregivers’ circumstances. Perceived safety and acceptability depended on this alignment and on effective organisational collaboration. Sustainable integration of HaH in GP practice requires clear communication pathways and greater involvement in decision-making. These findings provide practical guidance for implementing HaH and HaNH in acute care for older adults.

## Key Points

- Hospital at home and hospital at nursing home are meaningful alternatives to hospital care for older adults with acute moderate-to-severe respiratory illness.
- Care setting feasibility depends on contextual factors of patients and informal caregivers such as physical stability, availability and emotional readiness.
- Emphasising the importance of context-sensitive triage, involving patients and informal caregivers in shared decision-making.
- General practitioners emphasised that the intervention’s success depended on its compatibility with daily general practice.
- General practice feasibility was mainly shaped by organisational capacity, the intervention’s flexibility and the availability of time and resources.

## Introduction

Lower respiratory tract infections (LRTI), including pneumonia, are common and potential severe diseases in older adults [1]. However, its diagnosis can be challenging because older adults frequently present with atypical symptoms such as confusion or falls rather than classical respiratory signs. Although hospitalisation provides access to specialised diagnostics and treatment, it also carries substantial risks for frail older adults [2, 3]. Admission is associated with functional decline, loss of independence and reduced well-being, as well as iatrogenic harms such as delirium, falls, pressure ulcers and hospital-acquired infections [4–6]. Psychological distress from the unfamiliar environment and overstimulation, alongside suboptimal experiences in emergency settings, further contribute to negative outcomes [7, 8]. In addition, hospitalisation also places a burden on informal caregivers, who may face limited visiting options, travel demands and insufficient support [7]. In ageing societies such as the Netherlands, the growing number of older adults is accompanied by a higher burden of acute conditions requiring hospital care, including LRTIs. With limited capacity in nursing homes and community care, timely hospital discharge is often difficult, prolonging admissions and exacerbating strain on the healthcare system [9–12].

To address these challenges, alternative models of acute care are being explored. Hospital-at-home (HaH) is one such approach, providing treatments such as intravenous medication, oxygen therapy and daily monitoring in the home environment [4, 7, 8, 13, 14]. Technological innovations, including point-of-care diagnostics and digital monitoring, have expanded the feasibility of HaH. Evidence suggests that HaH can reduce costs and maintain or improve outcomes in older adults [9, 10, 14, 15]. Importantly, patients often value the comfort of familiar surroundings. Despite growing implementation, many evaluations have focused specifically on clinical outcomes, with less attention to patient satisfaction, caregiver burden and professional experiences [7–9, 16]. Since HaH relies on collaboration between multidisciplinary teams and support from informal caregivers, understanding these perspectives is essential for sustainable implementation.

With these challenges in mind, the integrated care pathway (ICP) ‘The Hague RTI Care Bridge’ was recently developed in an urban area in the Netherlands [17, 18]. This ICP was designed for community-dwelling older adults with moderate-to-severe LRTIs and aimed to provide a safe and person-centred alternative to hospital admission. It included three care pathways tailored to individual circumstances: (i) HaH with treatment and monitoring at home, (ii) Hospital-at-nursing-home (HaNH), in which older adults were temporarily admitted to a nursing home when treatment at home was not feasible and (iii) traditional hospital admission.

To ensure that our ICP meets the needs of patients and informal caregivers while remaining feasible for healthcare professionals, insight into their experiences is crucial. Although HaH models are increasingly implemented, research has mainly examined single care settings or specific stakeholder groups, often from hospital- or policy-led perspectives [19]. Limited attention has been paid to how HaNH is experienced as part of an ICP and to how general practitioners (GPs) experience their role in HaH. Therefore, this qualitative study aimed to explore experiences across the different care settings of ‘The Hague RTI Care Bridge’, from the perspectives of older adults, their informal caregivers and GPs involved in the HaH care pathway.

## Methods

### Setting and participants

This current qualitative study was embedded within a prospective mixed methods study designed to evaluate the ICP ‘The Hague RTI Care Bridge’ [17, 18]. Respondents for this qualitative study were selected using convenience sampling from the study population of the overarching mixed methods study [20, 21]. The first 12 included patients per care pathway (HaH, HaNH and hospital) who provided consent were also invited for an interview. For each included patient, an involved informal caregiver was also invited to participate. Informal caregivers were eligible if aged ≥18 years. Additionally, the 11 HaH GPs were invited for a separate interview, of whom 4 declined participation. Only GPs involved in the HaH pathway were interviewed, as this model introduced a new role for GPs in coordinating care at home. In the other pathways (HaNH and hospital admission), medical care (mostly) continued as part of usual care by elderly care specialists or hospital-based physicians [17].

### The Hague Airbridge ICP

The protocol of the overarching ICP consisted of three care pathways (i) HaH with treatment and monitoring at home, (ii) HaNH for patients unsuitable for home care and (iii) traditional hospital admission [18]. The study was conducted in an urban region and involved collaboration between two large home care organisations, acute home care, two city hospitals and a GP care group representing ~600 local GPs. The study of the new ICP ran from December 2022 to April 2024. The overall study population consisted of adults aged 65 years and older who presented at the emergency department (ED) with a clinical diagnosis of acute moderate-to-severe LRTI or were diagnosed by the GP. Defined by Pneumonia Severity Index (PSI) class ≥3 or Confusion, Urea, Respiratory rate, Blood pressure and age ≥65 years (CURB-65) score ≥2, an oxygen saturation ≥92% and a respiratory rate ≤24 breaths/minute with a maximum of 5 l of oxygen suppletion. Allocation to the different care pathways was guided by these predefined clinical and organisational criteria, pathway availability and informal caregiver support. Full inclusion and exclusion criteria are described in the published protocol [18].

#### Data collection

During the first visit by a research team member, a range of patient characteristics were collected, including the Charlson Comorbidity Index (CCI) [22]. Qualitative data were collected through semi-structured face-to-face interviews by a research team member, 2–3 weeks after inclusion of the study. Interviews were conducted by two interviewers: a female specialised geriatric nurse with no prior relationship to participants and no involvement in the intervention, and a male internal medicine resident who was involved in developing the pathway and had previously met some participants in clinical care. The interview guides were developed using the Consolidated Framework for Implementation Research (CFIR), which provides guidance to identify implementation determinants of an intervention [23–26]. We used the CFIR to explore the experiences of different stakeholders. Interview questions were informed by the five CFIR domains: intervention characteristics, outer setting, inner setting, characteristics of individuals and the implementation process [27]. Separate interview guides were developed for each respondent group per care pathway (patients, informal caregivers and GPs, see Supplementary Appendices S1, S2, S3). Interviews with patients and informal caregivers were mostly jointly conducted and took place at their residence or place of stay. All GP interviews took place at their offices.

#### Data analysis

Descriptive statistics were analysed using SPSS version 29. Interview data were analysed using Atlas.ti version 22 and a deductive thematic approach, as described by Braun and Clarke [28], guided by the CFIR. All interviews were conducted in Dutch, audio recorded, transcribed verbatim and pseudonymised. Quotations were translated into English by the primary analyst (I.V.), with attention to preserving contextual meaning, and checked by a second researcher to minimise interpretative bias. As part of the familiarisation phase, the primary analyst (I.V.) re-read all transcripts and re-listened to selected audio files to clarify unclear sections. The analysts were not involved in data collection and had no prior relationship with respondents. Interviews with patients and informal caregivers were analysed separately from GPs. All interviews were coded by the primary analyst, focusing on fragments relevant to the study aim. Constructs from the five CFIR domains were used to guide the coding process. To enhance coding consistency and rigour, a second coder (R.P.) independently coded eight transcripts (two per patient/informal caregiver pathway and two GP interviews). Coding was then reviewed jointly in in-depth comparison sessions, during which all coded quotations were discussed until consensus was reached and discussed with a third researcher (C.v.N.). When necessary, code definitions were refined and applied consistently in subsequent transcripts. Reflexivity was integrated throughout the analysis. Reflexive memos documented analytical decisions and contextual reflections. Regular meetings between co-researchers (I.V. and R.P.) supported critical reflection on coding decisions and theme development. Segments were organised per CFIR-construct and rarely used constructs (<15 fragments) were recoded into related, dominant constructs. Preliminary summaries were written per construct and discussed with the research team to identify relevant findings. Based on these discussions, overlapping findings in constructs were clustered, and overarching new themes outside of the CFIR domains were developed separately for the patient and informal caregivers and for the GPs.

#### Ethical considerations

The Medical Research Ethics Committee Leiden The Hague Delft confirmed that the Medical Research Involving Human Subjects Act did not apply to this study (reference number: N22.078). Ethical approval was granted by the Institutional Scientific Review Board of the leading hospital (reference number: T22-066). The study was registered in the ISRCTN registry (ISRCTN68786381). All respondents received oral and written information and provided written informed consent. All methods were performed in accordance with the Declaration of Helsinki of 2024.

## Results

### Characteristics of the population

A total of 42 interviews were conducted, involving 34 patients, 33 informal caregivers and 7 GPs. No information was collected regarding patients and GPs that declined to participate in the study. There were 12 patient/informal caregiver interviews in the hospital group, 12 patient/informal caregiver in the HaNH group, and 11 patient/informal caregiver and 7 GP interviews in the HaH group. An overview of interview attendance by care pathway and respondent type is presented in Table 1.

**Table 1:** Overview of interview attendance by patients, informal caregiver and general practitioners, stratified on care pathways.

Hospital		HaNH		HaH		
*ID*	*Present*	*ID*	*Present*	*ID*	*Present*	*Involved GP interviewed*
1	p + ic	13	p + ic	25	p + ic	Yes
2	p + ic	14	p	26	p + ic	Yes
3	p + ic	15	p + ic	27	ic (+p)^a^	Yes
4	p + ic	16	p + ic	28	ic	No
5	p + ic	17	p + ic	29	p + ic	Yes
6	p + ic	18	p	30	ic (+p)^a^	No
7	p + ic	19	p + ic	31	ic+^b^	Yes
8	p + ic	20	p + ic (+ic)^c^	32	p + ic	Yes
9	p + ic	21	p + ic	33	p + ic	Yes
10	p + ic	22	p + ic	34	p + ic	No
11	p + ic	23	p + ic	35	p + ic	No
12	p + ic	24	p + ic (+ic)^c^			

Abbreviations: p, patient; ic, informal caregiver.

^a^Patient present but did not actively contribute to interview.

^b^Patient died between study inclusion and interview date. Death was unrelated to the LRTI.

^c^Two informal caregivers present but interview primarily conducted with one informal caregiver.

Demographic characteristics of the patient population are stratified by care pathway (Table 2). The mean age in years was comparable between groups, with the highest average age observed in the HaNH group (81 ± 9), followed by the hospital group (78 ± 7) and the HaH group (78 ± 9). Most patients were male (66%), and the living situation varied considerably, with a higher proportion of patients living alone in the HaNH group (58%) compared to the HaH group (9%). None of the hospitalised patients had received home care prior to admission, in contrast to 25% of HaNH patients and 18% of HaH patients. Severe pneumonia (PSI class V) was most common in the hospital group, while not present in the HaNH group. Chronic obstructive pulmonary disease was particularly prevalent among hospitalised patients (75%), and approximately one-third of all patients had congestive heart failure or type 2 diabetes mellitus. Comorbidity burden, as measured by the CCI, was high across all groups (median 6). Mobility limitations were most frequently reported in the HaNH group (42%). Finally, median quality of life scores were highest in the hospital and HaH groups (80), and slightly lower in the HaNH group (73).

**Table 2:** Characteristics of patient population.

	HaH (*n* = 11)^a^	HaNH (*n* = 12)	Hospital (*n* = 12)	Overall (*N* = 35)
*Age in years*				
*Mean (*±*SD)*	78 (±9)	81 (±9)	78 (±7)	79 (±8)
*Gender*				
*Female n (%)*	4 (36)	6 (50)	2 (17)	12 (34)
*Living situation*				
*Alone n (%)*	1 (9)	7 (58)	3 (25)	11 (31)
*Prior home care*				
*Yes n (%)*	2 (18)	3 (25)	0 (0)	5 (14)
*Number of caregivers*				
*Median (*±*IQR)*	2 (2)	1 (2)	2 (2)	2 (2)
*PSI class*				
*III n (%)*	5 (46)	3 (25)	2 (17)	10 (29)
*IV n (%)*	4 (36)	9 (75)	7 (58)	18 (57)
*V n (%)*	2 (18)	0 (0)	3 (25)	5 (14)
*COPD*				
*Yes n (%)*	5 (46)	5 (42)	9 (75)	19 (54)
*CHF*				
*Yes n (%)*	4 (36)	3 (25)	4 (33)	11 (31)
*Diabetes mellitus*				
*Yes n (%)*	4/10 (40)	4/10 (40)	2/12 (17)	10/32 (31)
*CCI*				
*Median (±IQR)*	6 (3)	5 (3)	6 (3)	6 (3)
*Mobility (EQ-5D-5L)*				
*No problem n (%)*	5/10 (50)	4 (33)	8 (67)	17/34 (50)
*Slight problem n (%)*	2/10 (20)	1 (8)	2 (17)	5/34 (14)
*Moderate problem n (%)*	3/10 (30)	2 (17)	1 (8)	6/34 (18)
*Severe problem n (%)*	0 (0)	5 (42)	1 (8)	6/34 (18)
*QoL*				
*Median (±IQR)*	80 (25)	73 (20)	80 (20)	80 (20)

*COPD*, *constructive obstructive pulmonary disease; CHF*, *congestive heart failure; IQR*, *inter-quartile range; QoL*, *quality of life; SD*, *standard deviation.*

^a^ *Deceased patient included in baseline demographics.*

Informal caregivers (N = 33) had a median age of 68 years ranging from 64 years (HaNH) to 71 years (HaH). Most informal caregivers were female (73%), with the highest proportion in the hospital group (83%). The type of relationship to the patient varied across groups: in the hospital group, most were partners (67%), whereas in the HaNH group, children were most common (60%). The HaH group showed a more mixed distribution, with 55% as partners and 27% as children. All seven GPs were female, with a median age of 41 years (±7) and 12 years (±8) of experience working as a GP.

### Qualitative analysis of experiences

We identified three themes regarding experiences from the interviews with patients and informal caregivers: (i) matching care to needs, (ii) shared decision-making and communication and (iii) organisational collaboration. In addition, reflections on the overall facilitators and barriers of each care setting were identified and summarised. Offering insights into how each pathway was experienced in practice. The CFIR was used to explore experience facilitators and barriers. From patient and informal caregivers interviews the most used constructs were: compatibility, knowledge & beliefs, access to knowledge & information, engaging, executing and cosmopolitanism. Based on the GP interviews, two implementation-related themes were identified: (i) practical feasibility and (ii) engagement of GPs. These themes reflect the barriers and facilitators on how the intervention was perceived and integrated into daily general practice. From GP interviews, the most used constructs were: compatibility, available resources, organisational rewards & incentives, relative advantage, access to knowledge & information, engaging and cosmopolitanism.

### Experiences of patients and informal caregivers

#### Matching care to needs

The central theme emerging across all interviews concerned the perceived appropriateness between the care setting, the patient’s needs and caregiver’s needs in a broad context. This perceived appropriateness was shaped not only by the clinical condition but also by a range of non-clinical factors, including social environment, functional capacity, emotional wellbeing and practical feasibility.

##### Personal suitability

For patients, compatibility with a particular care setting was determined not solely by clinical considerations discussed by the doctor, but also by their own individual preferences, values and beliefs regarding what different care settings entail, such as maintaining independence, avoiding institutional care and prior experiences with the healthcare system. Crucially, this sense of fit also needed to extend to informal caregivers. Several informal caregivers reported that their own emotional resilience and functional and physical capacity influenced how appropriate they perceived a given care pathway to be (Table 3, Quote A).

**Table 3:** Patient and informal caregiver theme quotes.

Theme	Interviewee and care setting	Quote	Reference
Matching care to needs			
	Informal caregiver, hospital	*It could have worked, technically, but I’m a terrible hypochondriac. I would have been on the phone with the hospital constantly, saying things like: ‘Now he’s doing this. . . now that. . .’*	A
	Patient, hospital Patient, HaH	*It’s a good system, but it only works if there are two of you. You need a partner who’s able to help and who isn’t functionally illiterate.* *The mere fact that you don’t really have to worry about anything. Everything is well taken care of.*	B C
	Patient, HaH (transferred to hospital)	*I don’t know if the treatment would have been any different at home than in hospital, but at home I just couldn’t take care of myself. And my wife isn’t in the best shape either, so she didn’t really feel up to it. . . She can manage, but with how ill I was, she just didn’t feel comfortable. She’s been through a lot with me already. That made her very anxious.*	D
Shared decision-making and communication			
	Patient, HaH	*Yes, completely. ‘If it’s possible, we send you home, but you do need oxygen’. You know, I liked that. It felt right.*	E
	Informal caregiver, hospital	*It was never explained that there was a choice, whether to go from the emergency department to a) hospital, b) nursing home, or c) receive care at home. I didn’t know that was even an option.*	F
	Informal caregiver, hospital	*. . .when they explained something, they didn’t realise he’d forget it within an hour. I had to ask all the questions later, and he had no idea. Then I’d ask: has anyone been in? And he’d say: ‘No, no one came.’ But apparently two doctors had been by. That’s what I miss. I’m his partner, and I’m supposed to be his anchor. It’s about us, right?*	G
Organisational collaboration			
	Patient, HaNH	*They’re still in good contact. I heard from my GP that all the information from the hospital also reached them, and their information went back to the hospital as well. So, in that sense, I think the collaboration is good, as far as I can tell, of course.*	H
	Informal caregiver, HaNH	*Well, I found it [organisational collaboration] a bit disorganised. We had to make phone calls ourselves to sort things out. It wasn’t immediately clear who was responsible for what.*	I

##### Conditions for receiving care at home

Regarding care at home, respondents identified several preconditions that determined its feasibility. This model of care was generally regarded as suitable for patients with a stable physical condition, clinically uncomplicated and supported by an adequate informal care network. Patients needed to be mobile, no or mildly cognitively decline, and confident in managing basic aspects of self-care, while caregivers had to be available and prepared to assume a supervisory and supportive role (Table 3, Quote B).

In such circumstances, home-based care was viewed as feasible, appropriate and appreciated (Table 3, Quote C). However, when these conditions were not fulfilled, because of being physically weak, having memory and cognitive problems, or the absence of an informal caregiver, home treatment was perceived as unsafe or unmanageable. This was illustrated by one patient’s experience during the initial phase of HaH, which ultimately lead to hospital admission. He described how his symptoms, previous illness experiences and his wife’s emotional distress made coping at home difficult (Table 3, Quote D).

This theme emphasises the significance of context-sensitive triage processes that encompass not only clinical criteria but also the practical, emotional and relational dimensions of care.

#### Shared decision-making and communication

##### Involvement and communication in the care process

While considering contextual factors in triage, respondents emphasised the importance of being actively involved in decision-making. Across care settings, the extent of shared decision-making and the quality of communication between doctor and patient and caregiver influenced how safe, confident and supported patients and informal caregivers felt throughout the care process. From the patient and caregiver interviews, it became clear that the level of shared decision-making during triage varied between care settings. Respondents receiving home-based care described being consulted about their preferences and actively involved in choosing the care pathway. This fostered reassurance that the selected option aligned with their needs and values. One patient reported feeling supported and confident in the decision to receive care at home (Table 3, Quote E). Conversely, patients admitted to hospital or transferred to a nursing home often indicated that decisions were made unilaterally by healthcare professionals. The process was described as rushed or unexpected, with limited explanation or opportunity to express concerns. Consequently, some experienced these decisions as imposed rather than jointly agreed upon, leading them to question the appropriateness of the chosen setting and feel less in control of their care (Table 3, Quote F).

##### Communication during care

Beyond the triage phase, effective communication remained essential throughout the care process according to patients and caregivers. In the home-based setting, clear, step-by-step instructions enhanced patients’ sense of competence and control. In contrast, respondents in hospitals and nursing homes frequently described communication as incomplete, overly technical or inconsistent. This hindered patients’ ability to remain informed and involved, while informal caregivers were often even more excluded. One caregiver recounted lacking information because her partner had no recollection of what had been explained (Table 3, Quote G).

These experiences were shaped not only by individual interactions but also by the degree of collaboration between care organisations, explored further in the following theme.

#### Organisational collaboration

Patients and informal caregivers described varying experiences regarding how care providers, hospitals, nursing homes and home care services, collaborated across settings. In a few instances, respondents observed visible signs of cooperation, such as shared information or clear communication between providers. When this occurred, it contributed to a sense of clarity and continuity, as expressed by a nursing home patient (Table 3, Quote H). Although formal coordination may have been well organised in the background by protocol, it was often not visible to patients or their families. Patients and informal caregivers reported having to contact care providers themselves to clarify next steps or transfer information. This lack of transparency in coordination frequently led to confusion about responsibility and created a sense of insecurity (Table 3, Quote I). In addition to describing specific incidents, some respondents reflected more broadly on how collaboration between organisations might be improved for example, by strengthening communication channels or ensuring that care transitions are visibly followed up. These reflections suggest that collaboration should not only take place behind the scenes but also be made visible and comprehensible to patients and their informal caregivers.

#### Overall facilitators and barriers of each care setting

This section of the patient and informal caregiver interviews summarises the perceived facilitators and barriers of each care pathway beyond the themes mentioned above. These reflections illuminate the experiences of receiving care across different settings, shaped by practical and emotional factors such as professional proximity, autonomy and support. Respondents described making trade-offs between safety, independence and convenience.

##### Hospital admission: clinical safety and professional availability,  but busy surroundings

The hospital was primarily valued for its available medical services. Respondents felt reassured by the constant presence of medical personnel, the availability of diagnostic tools and the potential to detect other underlying conditions. Particularly in cases of severe symptoms, hospital care was perceived as the most appropriate and comprehensive option (Table 4, Quote J).

**Table 4:** Quotes of patients and caregivers regarding facilitators and barriers per care setting.

Facilitators and barriers of each care setting	Interviewee and care setting	Quote	Reference
	Patient, hospital	*The attention from the nursing staff and their professionalism. They really know what they’re doing, and that gave me a great sense of reassurance.*	J
	Informal caregiver, hospital	*A hospital ward like that is just too chaotic. It’s not pleasant. You know. . . it’s not the kind of place anyone would be happy to be in.*	K
	Patient, HaNH	*I think you’re looked after more closely in a nursing home than in a hospital. In hospital, you’re really left to yourself, I felt like I had to monitor everything myself. I find the environment in a nursing home more pleasant, it feels more homely. A hospital. . . well, it’s not exactly cheerful. Being able to get your own cup of tea here, that’s actually quite nice.*	L
	Informal caregiver, HaNH	*I was sceptical at first. You tend to think of hospital as the ideal, everything’s within reach, you press a button, and someone comes. And I guess it’s like they say: what you don’t know, you tend not to trust. But looking back, I think it was actually the best choice.*	M
	Informal caregiver, hospital	*We’ve had several family members in a nursing home. My own mother was there for twelve years, and my father-in-law too. So, we know what a nursing home is like. You can hear her say: ‘You never come out of here, you know. Hardly ever’.*	N
	Patient, HaH	*I felt like a person of royal blood. I had never experienced that much attention before*	O
	Informal caregiver, hospital	*I think it’s easier to talk when you’re at home and there’s a nurse around who knows you. In hospital, the nurses are always rushing past. I see them when I visit, but there’s no real contact.*	P

Informal caregivers also highlighted the relief provided by temporary respite from caregiving responsibilities and the emotional reassurance that the patient was under continuous professional supervision. However, this sense of safety was often tempered by discomfort with the hospital environment itself. The setting was described as overstimulating and impersonal, with shared rooms, limited privacy and frequent disturbances during day and night. Its institutional character was sometimes experienced as misaligned with personal comfort (Table 4, Quote K). Additionally, the hospital was occasionally perceived as unnecessarily intensive for managing moderate infections, particularly when alternative care options would have sufficed. Concerns were also raised regarding the risk of hospital-acquired infections and potential functional decline during admission.

##### HaNH: stability and safety, but a new experience

HaNH was perceived as a safe and structured alternative for patients unable to receive care at home. Patients valued the proximity of staff, the quiet atmosphere and the personalised attention. Compared with hospital care, many patients and informal caregivers described the environment as more homely and less intense, fostering a sense of security and connection (Table 4, Quote L). The presence of other residents and staff also provided social contact, which was often experienced as supportive. Like hospital care, informal caregivers appreciated the temporary relief from caregiving responsibilities. Some initially expressed scepticism regarding nursing home care but later acknowledged its benefits (Table 4, Quote M). Furthermore, nursing homes were associated with long-term dependency or permanent placement, leading to hesitation or emotional resistance at the time of admission. These perceptions were partly influenced by unfamiliarity with the short-stay function of the facility, reflecting broader societal views on nursing home care (Table 4, Quote N). At the same time, several barriers were reported. The absence of private facilities, such as individual bathrooms, was frequently described as inconvenient or undignified. In some cases, the facility’s distance posed a barrier for informal caregivers, who preferred a location closer to home to enable more frequent visits. However, all respondents who experienced this pathway described the nursing home setting positively. Many emphasised that the structured care, proximity of staff and social contact outweighed the challenges associated with the temporary change in setting.

##### HaH: autonomy, comfort and conditional feasibility

Home-based care was valued for allowing patients to remain in a familiar and comfortable environment. Being able to sleep in one’s own bed, maintain daily routines and avoid hospital or nursing home admission were frequently described as reassuring. The home was perceived as a quiet and private setting that supported both physical and emotional well-being. Several respondents noted that clear information on emergency procedures and the presence of a partner or family member provided an additional sense of security. The level of attention received from the GP and the care team at home was described as warm and personal (Table 4, Quote O). For some, care at home also enabled more personalised and calm interactions with professionals, compared with the hurried atmosphere in institutional settings (Table 4, Quote P). Despite these benefits, several challenges were identified. The presence of medical equipment, particularly portable oxygen systems, was burdensome due to size, operational noise and perceived safety risks. The setup and placement of devices were sometimes disruptive to the living environment. Additionally, the initial phase of HaH was unsettling for some, particularly when symptoms were severe, or the care process felt unfamiliar. Emotional stress was heightened by uncertainty about how to respond if the patient’s condition deteriorated, particularly for informal caregivers. In contrast, patients in HaNH experienced additional support and social contact, highlighting the different benefits offered by the two pathways.

### Experiences of general practitioners

#### Practical feasibility

GPs supported the principle of treating moderate-to-severe LRTIs outside the hospital but emphasised that the interven-tion’s success depended on its compatibility with general practice. Practical feasibility was mainly shaped by organisational capacity, the intervention’s flexibility and the availability of time and resources.

##### Mismatch between protocol and daily practice

Although the intervention was originally designed to enable direct referral to Ha(N)H from general practice, this did not occur in daily practice. Instead, patients were triaged via the ED, where the final decision regarding the care setting was made. GPs attributed this to logistical barriers, like time of day and busy schedules, and diagnostic uncertainty. While direct access to home treatment was considered desirable, several GPs noted that it was not feasible without hospital-based diagnostics (Table 5, Quote 1).

**Table 5:** Quotes of interviewed general practitioners regarding the hospital at home pathway.

Theme	Quote	Reference
Practical feasibility		
	*But as a GP, you don’t always have the tools to assess it yourself. So, you tend to think ‘let’s go through the emergency department instead’, at least they can do a chest X-ray there.*	1
	*Careful selection is key. The most important condition is that it fits the home situation and the patient’s needs.*	2
	*Time. And that’s exactly what we’re short of. Especially in the winter months, it’s just too busy.*	3
Engagement of GPs		
	*Information came to me indirectly. I didn’t get a clear leaflet or anything that a GP could use at a glance.*	4
	*I know better than the ED specialist what’s manageable for the patient and informal caregiver. I’d like to be involved in those decisions.*	5
	*I would have preferred to be involved earlier in the triage. As it was, it felt more like an announcement than a discussion.*	6
	*At the start, I felt the communication lacked structure. Sometimes it wasn’t clear who was in charge.*	7

##### Tailoring care to individual needs

GPs highlighted that a single, rigid protocol would be insufficient, as care provision should be adapted to the capacities and needs of both patients and informal caregivers. For some, one daily home care visit and a GP phone check-in were sufficient; others required daily GP visits and more intensive monitoring. The latter was indicated not always possible in daily practice. Flexibility also extended to the division of responsibilities. Some GPs preferred to remain closely involved in care delivery, whereas others delegated medical tasks to home care teams (Table 5, Quote 2).

##### Capacity and time constraints

Having to take care of one included patient in the study was not experienced as an increase in workload by the interviewed GPs. Feasibility was seen as dependent on balancing intervention-related duties with routine clinical work. Structural limitations, such as possible time constraints, limited staffing and fragmented coordination across organisations, were identified as key challenges, particularly during high-demand periods such as flu season or workforce shortages (Table 5, Quote 3). However, concerns were raised about potential strain if the number of HaH patients were to rise.

#### Engagement of GPs

GPs were generally receptive to the aims of the ICP, but their engagement depended on how well information, communication and shared responsibilities were organised across the pathway. Their inclusion in shared processes such as triage and follow-up shaped their sense of responsibility and trust in the intervention. Several GPs noted that relevant information about the ICP was not accessible through standard communication channels, such as the GP information system. In most cases, procedural details or contact information were communicated indirectly. Rather than lengthy background materials, GPs expressed a preference for concise, role-specific guidance presented through a centralised and easily accessible source, such as the Dutch GP treatment guidelines. The absence of structured access to such information was viewed as a barrier to timely and coordinated participation, particularly under time pressure (Table 5, Quote 4).

##### Coordination

Across interviews, GPs underscored the importance of being involved in decisions about whether a patient should be treated at home or temporarily admitted to a nursing home. They stressed that GPs often have deeper insight into a patient’s living situation, informal caregiver capacity and what is feasible within their general practice. While they did not expect exclusive decision-making authority, GPs described a professional duty to contribute expertise on what is appropriate and safe (Table 5, Quote 5). In several cases, GPs were informed of the inclusion only after the decision had been made, which limited opportunities for coordination (Table 5, Quote 6).

##### Communication

GPs indicated that effective participation relied not only on access to information but also on well-functioning communication with hospital and home care professionals. Some described close working relationships that enabled quick coordination and collaborative problem-solving, whereas others experienced more fragmented and ad hoc contact. In such cases, vague responsibilities and the absence of structured handover procedures impeded coordination (Table 5, Quote 7). GPs emphasised that clearly defined roles, timely updates and consistent communication are essential for effective collaboration and safe care planning.

## Discussion

This study provides a nuanced understanding of how older adults in two alternative acute care pathways, HaH and HaNH, and their informal caregivers, as well as GPs experienced the transformation of acute respiratory care according to one ICP, ‘The Hague RTI Care Bridge’. By examining all these experiences across multiple care settings within a single ICP, this study adds novel insights into the needed practical conditions and emotional readiness that shape feasibility, appropriateness and trust in provided care. Guided by the CFIR, the analysis identified facilitators and barriers influencing implementation during the pilot phase. After analysing all constructs used during coding, three new overarching themes were identified, corresponding to the experiences gathered from patient and caregiver interviews: *matching care to needs*, *shared decision-making and communication*, and *organisational collaboration.* In addition, there were *facilitators and barriers* of each care setting. Two themes were identified from GP interviews: *practical feasibility* and *GP engagement*.

During the analysis of the patient and caregiver interviews difficulty was experienced by the research team using the CFIR framework for evaluating the intervention by the recipients. CFIR constructs were used but new themes were formed beyond the five CFIR domains. Recent literature shows that the CFIR may not be the most appropriate framework to use [29]. Although allocation to the different care pathways was guided by predefined clinical and organisational criteria, our findings show that the perceived appropriateness of each care setting extended beyond these formal criteria to encompass the patient’s physical, emotional and social context. Moreover, this alignment had to be feasible for informal caregivers, who sometimes found home-based care unmanageable or unsafe due to anxiety or previous experiences. Although guidelines and reviews have acknowledged functional and social criteria for HaH eligibility, less attention has been given to how emotional strain or caregivers’ doubts may affect feasibility [30]. Earlier studies have described similar challenges in the context of acute illness, including pneumonia, though their focus lies on caregiving experiences *within* HaH rather than on how care setting appropriateness is assessed [31, 32]. Our findings add nuance by showing that doubts about feasibility can influence care setting choice, even when eligibility criteria appear to be met. Collectively, these results emphasise the importance of context-sensitive triage, ideally involving patients and informal caregivers in shared decision-making.

Respondents treated at home often felt actively consulted, whereas others, particularly those admitted to hospital or nursing home care, described the decision-making process as rushed or imposed. This resonates partly with findings from a recent study which reported that shared decision-making increases trust and acceptance of HaH care [33]. Beyond triage, respondents also highlighted the importance of clear and consistent communication during treatment. Similar results were found that caregivers value clarity but often feel excluded or overwhelmed [31, 34]. Our results extend these insights by showing that communication dynamics differed markedly between settings: home-based care fostered closer collaboration and step-by-step explanations, whereas institutional care was often more fragmented. Maybe this closer collaboration and communication was because the HaH pathway was completely new in our region.

Such differences reflect a broader issue: communication difficulties frequently mirror challenges in organisational collaboration [35, 36]. Respondents reported varying experiences of coordination between care providers. When collaboration was visible, it fostered a sense of continuity and safety. However, many patients and informal caregivers remained unaware of how professionals worked together behind the scenes. This aligns with findings of earlier research which noted that despite substantial coordination efforts, patients often lacked insight into how these processes unfolded [33]. Similarly, other studies observed that unclear transitions and the absence of visible communication can lead to confusion and insecurity [35, 36]. While those studies mainly addressed post-discharge or planned hospital-to-home transitions, our findings extend the perspective to acute illness, illustrating that even during short-term episodes, visible organisational collaboration can influence the sense of safety and coordination.

Alongside implementation-related themes, patients and their caregivers also shared descriptive reflections on what each care setting offered in terms of comfort, autonomy and support. They weighed constant professional supervision against the familiarity of home. These findings align with prior research showing that patients appreciate the calm and privacy of home-based care but may feel uncertain when professional presence is limited [7, 9, 13]. Conversely, both our study and others described hospital environments often as medically reassuring but emotionally draining due to noise, lack of privacy and feelings of detachment and sometimes even potentially harmful [34, 37]. Our study confirmed these patterns and added new insights into how temporary nursing home care is perceived, particularly the unfamiliarity surrounding short-stay functions and the emotional connotations associated with nursing homes.

All included GPs were female, which may have resulted in a more one-sided perspective. However, this aligns with the current Dutch GP workforce, which is predominantly female [38]. GPs reported comparable challenges as patients concerning triage, communication and organisational coordination. While they supported the principle of treating moderate-to-severe LRTIs outside the hospital, successful implementation will depend on how well the intervention integrates into general practice. Practical feasibility was influenced by time pressure, limited diagnostic tools and the flexibility to tailor care. Engagement depended on timely access to information, involvement in triage decisions and coordination with hospital and home care teams. Similar preconditions for successful collaboration have been noted in previous work, though most studies have examined HaH implementation from a hospital- or policy-led perspective [19]. Our study contributes by illuminating GPs’ experiences within a regional intervention, showing that even well-defined HaH and HaNH protocols may fail to translate into workable practice and patients end up at the ED. Research on transitional care has also shown that GPs often lack timely access to relevant information, particularly when hospital-based decisions occur without prior consultation [39]. Our findings reinforce these observations and further highlight that GP engagement was hindered by unclear responsibilities and fragmented communication structures. Ultimately, the findings demonstrate that implementing out-of-hospital care successfully requires not only clinical readiness but also organisational coherence, culture change, clear communication between all involved parties, and sustained engagement of both professionals and informal caregivers.

### Strengths and limitations

This study incorporated the perspectives of patients, informal caregivers and GPs across three care pathways, addressing a gap in existing literature that often focuses on a single perspective or setting. By explicitly including informal caregivers, GPs and the nursing home group, it offered a more comprehensive understanding of experiencing and delivering out-of-hospital care. The substantial number of patient and informal caregiver interviews enabled in-depth exploration across perspectives. Most interviews with patients and informal caregivers were conducted jointly, which may have constrained the independent expression of caregiver perspectives but also reflected real-life dynamics and facilitated shared meaning-making. Analysis indicated that the final interviews within each pathway did not generate additional themes beyond those already identified, suggesting thematic completeness. The use of the CFIR provided structure that enhanced rigour and supports transferability to similar healthcare contexts, though this predefined framework may have limited flexibility to explore themes beyond its scope.

Another limitation is that not all GPs involved in HaH participated, which may have led to limited information. While the interview guide was not adapted during data collection, the CFIR-based structure, dual coding and regular team discussions strengthened analytical consistency. Reflexivity and a second coder further enhanced the confirmability of the findings. In hindsight, we also should have interviewed the elderly care physicians regarding the new care pathway and their involvement of treating these patients.

## Conclusion

This study contributes to growing evidence that Hospital at Home and Hospital at Nursing Home can serve as viable and meaningful alternatives to hospital admission for older adults with acute moderate-to-severe respiratory illness. Successful and positive experiences, however, extend beyond clinical eligibility and depend on how effectively care delivery aligns with the physical, emotional and social circumstances of patients and their informal caregivers. Perceived safety, comfort and acceptability were strengthened by context-sensitive triage, shared decision-making and clear interprofessional communication between care providers. Feasibility further relied on adequately supporting GPs to incorporate care pathways in their daily practice and active engaging of GPs. Our findings highlight the need for further structuring transmural professional collaboration, matching care to the needs of those involved and proactive support for informal caregivers in their evolving role or involving their social network.

## Supplementary Material

Supplementary_materials_afag144
