# Supplementary material for: Hospital-at-home and beyond: experiences of patients, caregivers and general practitioners with out-of-hospital care for moderate-to-severe lower respiratory tract infections in older adults—a qualitative study
Source: Age Ageing. 2026 Jul 10;55(7):afag144. doi: 10.1093/ageing/afag144 (PMC13354058; doi:10.1093/ageing/afag144)
Supplement: Supplementary_materials_afag144 [file supplementary_materials_afag144.zip › Supplementary_materials_afag144_Appendix3.docx]

**Appendix 3**

**Questionnaire (Interview Guide for Patients)**

**- First of all, do you agree that we record this conversation and that any quotes used will not be traceable to you personally?**
(The recordings will be deleted immediately after transcription, and the transcripts will be stored securely within Haga Hospital.)

**Intervention Characteristics**

- What did the home care you received consist of?

**Characteristics of Individuals**

- Did you feel that you received the care you needed?
  - Why or why not?
- To what extent were your needs and preferences taken into account when deciding whether you would be treated at home or in hospital?
- Was the treatment you received clear to you?
  - Were the reasons why this care was chosen for/with you clear?
- How was the option of home treatment communicated to you?
  - Was it clear what was expected of you?
  - Was it clear where you could turn for help?
- Did you feel safe and sufficiently supported during your home treatment?
  - What made you feel safe or unsafe during the home treatment?
- Were you ever afraid during the home treatment?
  - If yes, what made you feel that way?
  - If yes, do you think this would have been different if you had been admitted to hospital?
- Do you think providing this hospital treatment at home is a good option?
  - Why or why not?
  - Would you recommend this hospital treatment at home to others?

**Process**

- How did you find using the pulse oximeter and thermometer?
  - Was the explanation from the community nurse clear?
  - How could this explanation be improved?
- Do you think any adjustments are needed?
  - If so, what could make it better?

**Outer Setting**

- Do you see this collaboration between the GP, hospital, elderly care and home care as an improvement?
  - Why or why not?
- Did you feel that the collaboration went well?
  - Could you give an example?
- Did you feel that some things did not go well?
  - If so, what do you think went wrong?
