# Supplementary material for: Hospital-at-home and beyond: experiences of patients, caregivers and general practitioners with out-of-hospital care for moderate-to-severe lower respiratory tract infections in older adults—a qualitative study
Source: Age Ageing. 2026 Jul 10;55(7):afag144. doi: 10.1093/ageing/afag144 (PMC13354058; doi:10.1093/ageing/afag144)
Supplement: Supplementary_materials_afag144 [file supplementary_materials_afag144.zip › Supplementary_materials_afag144_Appendix1.docx]

**Appendix 1**

**Questionnaire (Interview Guide for GPs)**

**- First of all, do you agree that we record this conversation and that any quotes used will not be traceable to you personally?**
(The recordings will be deleted immediately after transcription, and the transcripts will be stored securely within Haga Hospital.)

**Intervention Characteristics**

- How did you hear about ***‘The Hague Respiratory Tract Infection Care Bridge*’?**
  - Was it clear to you from the start what it involved and what the options were?
- Do you think the care pathway for home treatment of older adults with an acute upper respiratory infection or pneumonia is a good option in the Haaglanden region?
  - To what extent do you think the GP culture in the Haaglanden region is open to this care pathway?
  - To what extent do you expect to use the pathway yourself in the future?
  - To what extent do you expect your colleagues to use the pathway?
- Did you feel safe and sufficiently supported during the home treatment of your patient?
  - What made you feel safe or unsafe during the home treatment?

**Inner Setting**

- To what extent does this care pathway align with existing structures and processes in care?
- Through which route did you initiate the care pathway for this home-treated patient?
  - What did the home care your patient received consist of?
- What is your perception of the support provided?
  - Were you able to find the relevant phone numbers easily?
  - Did you use the website?
  - How did you experience the communication with the internists/pulmonologists (if applicable)?
- What did you think of the home monitoring with the pulse oximeter and thermometer?

**Outer Setting**

- Do you see this collaboration between GPs, the hospital, elderly care and home care as an improvement?
  - Why or why not?
- Did you feel that the collaboration went well?
  - Could you give an example?
- Did you feel that some things did not go well?
  - If so, what do you think went wrong?
- What do you think should be changed to improve home treatment?
  - Why?
- What is currently going well in the home treatment and should definitely be maintained?
  - Why?
- Do you think it is a good option to provide this hospital treatment at home?
  - Why or why not?
- Would you recommend offering this hospital treatment at home to colleagues?
- Would you recommend this hospital treatment at home to patients?
  - For which other conditions do you see hospital treatment at home as an option?
- What potential barriers do you see to participating?
  - Both for healthcare professionals and for patients?
- Do you feel that you are adequately financially supported to provide this care?

**Characteristics of Individuals**

- Do you consider hospital treatment at home to be an essential option to mention to patients?
- To what extent did you feel that the patient received the care he or she needed?
  - Why?
- Do you think that patients’ preferences can be sufficiently taken into account?
  - Does this care pathway mainly meet the wishes of patients or of healthcare providers?
- What could be reasons to use or not to use the care pathway?

**Process**

- How did you find the process of initiating the care pathway in practice?
  - Which materials (website, leaflet, etc.) did you use to start it?
  - How much time did the start-up take?
- How could we improve the process of starting the care pathway?
  - Are there enough materials (websites, leaflets, etc.) available to use this care pathway?
